# Structural ensembles reveal intrinsic disorder for the multi-stimuli responsive bio-mimetic protein Rec1-resilin

**DOI:** 10.1038/srep10896

**Published:** 2015-06-04

**Authors:** Rajkamal Balu, Robert Knott, Nathan P. Cowieson, Christopher M. Elvin, Anita J. Hill, Namita R. Choudhury, Naba K. Dutta

**Affiliations:** 1 Ian Wark Research Institute, University of South Australia, Mawson Lakes campus, Mawson lakes, South Australia 5095, Australia; 2 ANSTO, Private Mail Bag, Kirrawee, New South Wales 2232, Australia; 3 Centre for Synchrotron Science, Monash University, Victoria 3800, Australia; 4 CSIRO Agriculture, Level 6, Queensland Bioscience Precinct, St Lucia, Queensland 4067, Australia; 5 CSIRO Manufacturing, Clayton, Victoria 3168, Australia

## Abstract

Rec1-resilin is the first recombinant resilin-mimetic protein polymer, synthesized from exon-1 of the Drosophila melanogaster gene CG15920 that has demonstrated unusual multi-stimuli responsiveness in aqueous solution. Crosslinked hydrogels of Rec1-resilin have also displayed remarkable mechanical properties including near-perfect rubber-like elasticity. The structural basis of these extraordinary properties is not clearly understood. Here we combine a computational and experimental investigation to examine structural ensembles of Rec1-resilin in aqueous solution. The structure of Rec1-resilin in aqueous solutions is investigated experimentally using circular dichroism (CD) spectroscopy and small angle X-ray scattering (SAXS). Both bench-top and synchrotron SAXS are employed to extract structural data sets of Rec1-resilin and to confirm their validity. Computational approaches have been applied to these experimental data sets in order to extract quantitative information about structural ensembles including radius of gyration, pair-distance distribution function, and the fractal dimension. The present work confirms that Rec1-resilin is an intrinsically disordered protein (IDP) that displays equilibrium structural qualities between those of a structured globular protein and a denatured protein. The ensemble optimization method (EOM) analysis reveals a single conformational population with partial compactness. This work provides new insight into the structural ensembles of Rec1-resilin in solution.

Genetically engineered biomimetic proteins that display responsiveness to external stimuli are emerging as a promising class of biomaterial for a wide range of applications including controlled drug delivery, regenerative medicine, biosensors, templating agents, and hybrid nanodevices^1^,^2^,^3^. Recent advances in biosynthesis and cloning techniques have created new opportunities in construction of a diverse library of peptides that are reproducible at the molecular level^4^. Recombinant protein synthesis techniques provide an attractive platform for the development of new soft materials with precise control over the composition (monomer sequence), size, structure and functions^5^,^6^. Several biomimetic protein polymers including variants of green fluorescent protein^7^, elastin^8^, resilin^3^,^9^ and abductin^10^ have been reported in the literature highlighting numerous features including responsiveness, organization, and functionality.

Native resilin is a cross-linked elastomeric extracellular matrix protein found in many arthropods. It has long been recognized for its remarkable resilience (>92%), high fatigue life (in excess of 300 million cycles), and its role in the jumping, flying and sound production mechanisms in insects^11^. The identification of the gene sequence of *Drosophila melanogaster* resilin by Ardell and Andersen in 2001 opened new routes to engineer resilin-mimetic polypeptides (RMPs)^12^,^13^. Owing to the low stiffness, high fatigue lifetime, resilience, and attractive biochemical and mechanical properties, cross-linked RMPs have emerged as valuable materials for biomedical applications including tissue engineering^3^,^14^,^15^,^16^,^17^. Rec1-resilin, encoding the N-terminal domain (exon 1) in native *D. melanogaster* resilin (CG15920 gene product from transcript CG15920-RA) was first reported by Elvin *et al.*^18^ Rec1-resilin is a water soluble RMP and consists of 310 amino acid residues with 18 copies of a 15-residue repeat consensus sequence: GGRPSDSYGAPGGGN (Fig. 1 in Supplementary Information)^13^,^18^. Rec1-resilin exhibits multi-stimuli responsiveness including thermal, pH, ion, and photo responsiveness in aqueous solution^19^. It also displays unusual dual phase thermal transition behaviour, i.e. upper critical solution temperature (UCST) as well as lower critical solution temperature (LCST)^19^. Potential uses of multi-stimuli responsive Rec1-resilin have been demonstrated by creating patterned surfaces^20^, responsive interfaces^21^ and template-directed synthesis of controlled noble metal nanoparticles^22^,^23^. Cross-linked Rec1-resilin hydrogels exhibit near-perfect rubber-like elasticity with outstanding resilience (>92%) and negligible creep behaviour^18^,^24^. The near-ideal rubber-like elasticity of Rec1-resilin has been reported to be entropic in origin^24^. The molecular and structural basis of the characteristics of Rec1-resilin in solution and of the cross-linked Rec1-resilin hydrogels has not yet been fully elucidated.

Based on limited experimental evidence, the current hypothesis is that the properties of Rec1-resilin arise from its unique molecular composition, high degree of conformational dynamics, and its ability to interact with the environment with a high level of specificity^18^,^19^,^20^,^21^,^22^,^23^,^24^,^25^,^26^. Early studies of cross-linked native resilin from insects using wide angle and small angle X-ray diffraction and electron microscopy noted the amorphous nature of resilin^27^. Despite early and subsequent work, quantitative information on the intrinsic structure of Rec1-resilin remains elusive.^26^ The apparently disordered nature of Rec1-resilin precludes the use of many classical approaches to structural investigation^28^. Gaining structural and functional information about intrinsically disordered proteins (IDPs) presents well documented challenges^29^. Herein, we combine computational and experimental methods to examine the structural ensembles of Rec1-resilin in aqueous solution. We present a comprehensive description of the equilibrium structural ensembles for Rec1-resilin, which underpins the molecular basis of its properties.

## Results

### Secondary structure prediction

There has been significant progress towards accurate and reliable prediction of protein secondary structure from the primary amino acid sequence^28^,^29^. The secondary structure of Rec1-resilin was predicted from its primary amino acid sequence (Fig. 1 in Supplementary Information) using different key secondary structure modelling routines including DSC (Discrimination of protein Secondary structure Class)^30^, PHDsec (Profile network prediction HeiDelberg secondary structure)^31^, and SOPMA (Self-Optimized Prediction Method with Alignment)^32^. Each of these algorithms predicted a largely disordered structure. The predicted secondary structure was largely random coil (>85%) with some extended strand and a few helix configurations (Table 1). The PONDR^®^ (Predictor of Naturally Disordered Regions)^33^ algorithm fit suggested that the entire region of the protein Rec1-resilin is naturally disordered, with the disorder disposition value exceeding a threshold of 0.5 throughout the residue index (Fig. 2 in Supplementary Information).

### Circular Dichroism (CD) spectroscopy

**Figure 1:**
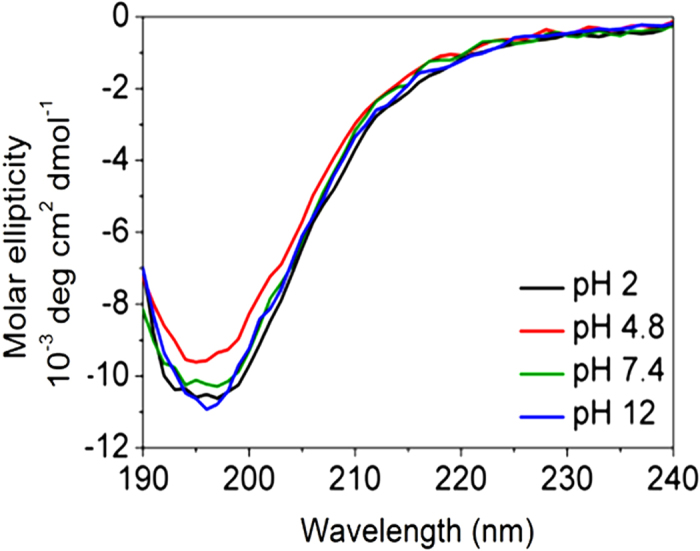
Far-UV CD spectra of Rec1-resilin measured as a function of solution pH. Rec1-resilin displays the minimum at around 196 nm, characteristic of a random coil secondary structure.

In order to gain greater insight into the secondary structure of Rec1-resilin, an aqueous solution of the protein was examined experimentally using CD spectroscopy. CD spectroscopy was performed over a wide range of pH. At physiological pH of 7.4, the measured far-UV CD spectra of Rec1-resilin (Fig. 1) displayed a large negative band with a single minimum at ~195-200 nm (due to π−π* transition) and a very low ellipticity above 210 nm. This observation suggested overall random coil characteristics of the protein^34^ and supports the theoretical assessments obtained via protein secondary structure prediction routines (Table 1). A quantitative estimation of the secondary structure(s) was attempted from the CD spectra using secondary structure deconvolution fits. CD deconvolution fit results using different algorithms respectively, CONTIN^35^, SELCON^36^, and CDSSTR^37^ with basis sets (containing some denatured and disordered soluble model proteins), indicate that Rec1-resilin is largely disordered (54.6-57.3%) at physiological pH with some contributions from helix (1-5.8%) and β-sheets and/or turns (38.4-41%) (Table 2). The quantitative difference between theoretical prediction and experimental data may be attributed to the maximum prediction accuracy of ~70% reported for the secondary structure modelling routines used^30^,^31^,^32^.

Rec1-resilin has an isoelectric point (IEP) of pH 4.8, and the protonated and/or de-protonated states of tyrosine amino acid residues (pKa~10.5) in the structure have been shown to affect the photophysical properties of Rec1-resilin in different pH environments^19^. The secondary structure of Rec1-resilin was analysed as a function of pH, and the CD spectra are presented in Fig. 1. Figure 1 shows a marginal reduction in ellipticity of Rec1-resilin on reduction of pH from 7.4 to the IEP (pH ~4.8) or below the IEP (Table 2) without any substantial secondary structure change. Moreover, only a slight secondary structure change was observed with increase in pH to 12 (Table 2). This minor change in CD spectra as a function of pH may be related to the change in surface charge on the Rec1-resilin without any appreciable secondary structural change^19^.

### Small Angle X-ray Scattering (SAXS)

**Figure 2:**
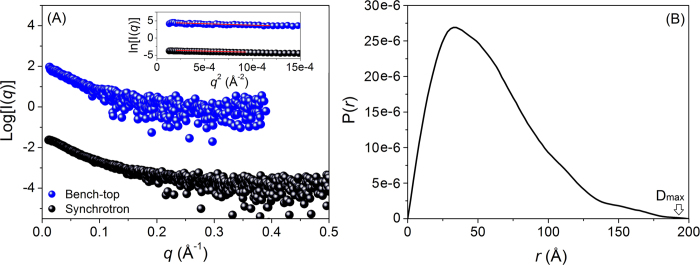
(**A**) Comparison of experimental SAXS patterns of Rec1-resilin collected from both bench-top (blue) and synchrotron (black) beam lines. Inset is the corresponding Guinier approximation plot used to determine the radius of gyration (*R*_*g*_) of the molecule. (**B**) Pair-distance distribution function, P(*r*), of Rec1-resilin derived from synchrotron SAXS data fit using PRIMUS program — asymmetric P(*r*) curve (characteristic of elongated molecule) with a maximum particle size (D_max_) estimated at ~200 angstrom.

**Figure 3:**
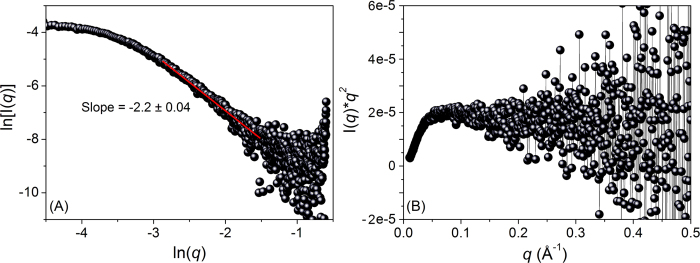
(**A**) The Porod plot and (**B**) Dimensionless Kratky plot of Rec1-resilin derived from synchrotron SAXS data. Rec1-resilin displays the characteristics of a partially compact molecule in-solution with estimated Porod slope (−2.2 ± 0.04) between that of Gaussian chains (~2) and collapsed polymer coils (~3). The Kratky plot displays an initial monotonic increase in the lower *q*-region followed by a plateau with gentle negative slope in the higher *q*-region — the characteristics of a non-folded overall random coil secondary structural conformation.

**Figure 4:**
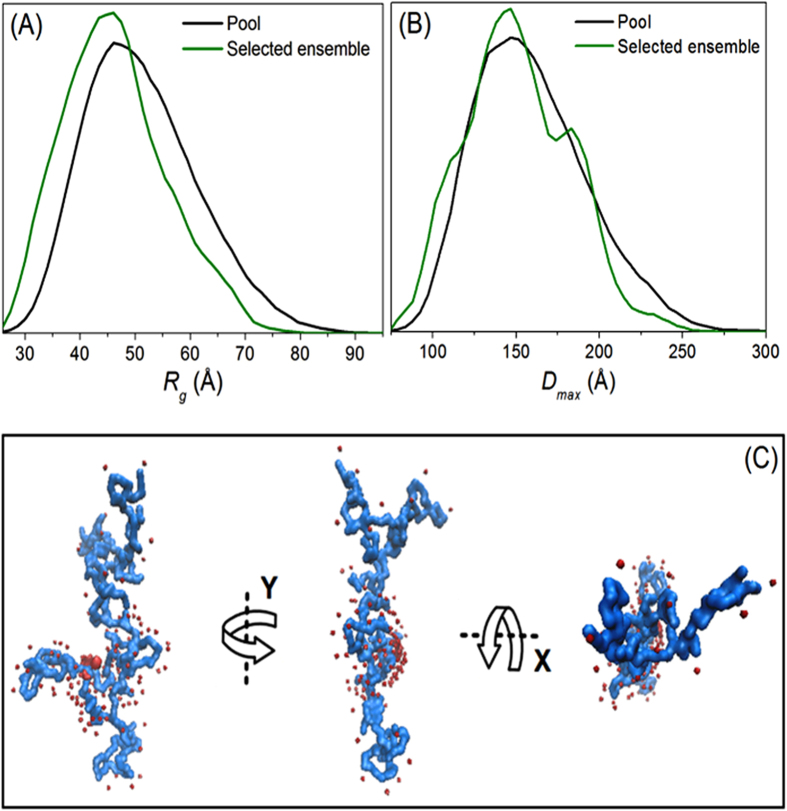
(**A**) Radius of gyration (R_g_) and (**B**) Maximum particle size (D_max_) distributions of Rec1-resilin plotted as functions of frequency (arb. unit) using the ensemble optimization method (EOM). (**C**) Representative *ab initio* 3D-model structure (one among an infinite ensemble of possible 3D-densities) of Rec1-resilin reconstructed using the GASBOR program from the distance distribution function output.

SAXS has been an indispensable technique over the last decade for addressing many of the fundamental structural questions of IDPs^38^,^39^. SAXS investigation of Rec1-resilin was carried out at physiological pH (~pH 7.4) using both a laboratory X-ray source and a synchrotron light source. Figure 2A shows the scattering cross section of 0.1% (i.e. 1 mg/ml) Rec1-resilin in solution from both synchrotron beam lines and bench top SAXS. The curves illustrate the scattering intensity I(*q*) as a function of the scattering vector (*q*)^40^:
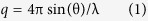
where, 2θ is the angle of scattering and λ is the wavelength of the X-ray. An investigation of synchrotron radiation damage to Rec1-resilin was conducted over a long period of time (~3h), and no change in the scattering cross section with time confirmed structural stability (Fig. 3 in Supplementary Information). It was also observed that a change in the Rec1-resilin concentration in solution from 0.013 to 0.1% promotes no inter-particle aggregation (Fig. 4 in Supplementary Information). With no effect of radiation damage, aggregation, or inter-particle interference observed (Figs. 3 & 4 in Supplementary Information), the scattering intensities were extrapolated to zero concentration in order to obtain model-independent structural information of the protein using the Guinier approximation^40^:
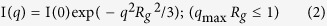
where, I(*q*) is the intensity of scattering, I(0) is the intensity at zero scattering and *R*_*g*_ is the radius of gyration. *R*_*g*_ represents the overall size of the protein at equilibrium conformation and was determined (using the PRIMUS program^41^) from the slope of the Guinier plot (Fig. 2A inset in main text & Fig. 4 inset in Supplementary Information). The *R*_*g*_ values of the 0.1% solution of Rec1-resilin were calculated to be ~43.4 ± 0.8 and ~43.3 Å ± 1.3 using synchrotron and bench-top SAXS data, respectively. The *R*_*g*_ values do not change for compositions in solution from 0.013, 0.025, 0.05, to 0.1% Rec1-resilin in solution (Fig. 4A inset in Supplementary Information).

The molecular mass (MM) of Rec1-resilin was calculated from the synchrotron SAXS data for the 0.1% Rec1-resilin solution using equation 3^42^:
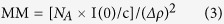
where, *N*_*A*_ is Avogadro number, I(0)/c is the forward scattering normalized against concentration, and ∆*ρ* is the product of scattering length density and partial specific volume of the protein. From the primary amino acid sequence of Rec1-resilin (Fig. 1 in Supplementary Information), the scattering length density and partial specific volume were estimated to be 3.399 × 10^−6^ Å^−2^ and 0.695 cm^3^ g^−1^ respectively using the web applet MULCh (http://smb-research.smb.usyd.edu.au/NCVWeb/input.jsp). The molecular mass of Rec1-resilin from SAXS was calculated to be 28.1 kDa with an oligomerization state ratio of 0.986 (experimentally measured molecular mass divided by theoretical molecular mass). The calculated molecular mass of Rec1-resilin from SAXS data was further validated with an experimentally measured molecular mass of 28.5 kDa using matrix-assisted desorption/ionization time-of-flight (MALDI-TOF) mass spectroscopy (Figure 5 in Supplementary Information). This is in agreement with the monomer molecular mass of 28.492 kDa reported by Elvin *et al.* and confirms that the protein has not suffered any degradation during purification^18^. Therefore, the high *R*_*g*_ measured for Rec1-resilin is not due to it being a stable multimer in solution. Though, MALDI-MS cannot resolve this unambiguously because a multimer might be dissociated during sample matrix preparation, but it certainly does not show ions that would correspond to multimeric species. The measured (using dynamic light scattering technique) hydrodynamic diameter (*D*_*h*_) of 0.1% Rec1-resilin at physiological pH (Figure 6 in Supplementary Information) was observed to be ~10.57 nm. Generally, the *R*_*g*_ value of an IDP protein is compared to its hydrodynamic radius (*R*_*h*_) to assess any residual structure^43^. For globular proteins, the compact structure yields
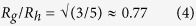


For denatured proteins the *R*_*g*_/*R_h_* ratio is approximately 1.40^43^,^44^. The experimental *R*_*g*_/*R_h_* ratio for 0.1% Rec1-resilin in solution at pH 7 is ~0.82. Although not quantitative, this ratio indicates the presence of residual structure in Rec1-resilin (*molten globule* and *premolten globule*)^44^. The bench-top SAXS data for 0.5 and 1.0% Rec1-resilin in solution gave *R*_*g*_ values of ~47.9 and ~50.9 Å, respectively (Fig. 4B inset in the Supplementary Information). The increase in *R*_*g*_ values for compositions above 0.1% Rec1-resilin in solution could be due to possible inter-protein interaction with increased protein concentration. For this reason the 0.1% Rec1-resilin synchrotron SAXS data were used for further analysis and modelling.

For a protein molecule consisting of 310 amino acid residues, the *R*_*g*_ value is expected to be around ~59.5 Å for an unfolded structure (based on chemically unfolded proteins with random coil behaviour) following a power-law relationship between the polymer length and the ensemble average *R*_*g*_ (equation 5)^45^:
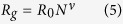
where, *N* is the number of monomers in the polymer chain (310), *R*_*0*_ is a constant (1.33 Å) that is a function of the persistence length of the polymer, and *ν* is the exponential scaling factor (0.588). For a compact structure (based on small α-helical globular proteins), the *R*_*g*_ value is predicted to be around ~19.6 Å^46^. A comparison of the experimentally observed *R*_*g*_ value (~43.4 Å) and the expected *R*_*g*_ values for unfolded and globular proteins using predictive tools, leads to an estimate that the structure of Rec1-resilin is largely unfolded but not completely disordered in nature^45^,^46^.

To determine the equilibrium molecular size and shape of uncrosslinked purified Rec1-resilin in solution, the pair-distance distribution function, P(*r*), as a histogram of all of the inter-atomic distances (*r*), was calculated by inverse Fourier transform of the scattering intensity, I(*q*)^47^. P(*r*) is considered to be more appropriate for *R*_*g*_ calculations of IDPs than Guinier’s approximation; because P(*r*) is a model independent function and requires the entire scattering spectrum. A similar approach has been adopted by Bernado *et al.*^48^ to describe an unfolded chain, where Guinier’s law is less appropriate and often underestimates the *R*_*g*_ values of extended chains. The shape of the P(*r*) distribution curve with asymmetric and extended tail region (Fig. 2B) suggests that Rec1-resilin is an elongated molecule in solution with a real space *R*_*g*_ ~47.8 Å, and a maximum molecular dimension (D_max_) ~200 Å (calculated using PRIMUS program^41^)^49^. The *R*_*g*_ value of Rec1-resilin is estimated to be ~50.7 Å using the Flexible-Meccano (FM) program proposed by Bernado *et al.*^38^ Using these values of *R*_*g*_ results in revised *R*_*g*_/*R_h_* ratios from 0.90 to 0.96. These observations support the view that Rec1-resilin is more compact than if it were a chemically denatured protein and is an IDP.

The fractal dimension of the scattering molecule, Rec1-resilin, was evaluated from the slope of the Porod plot (ln[I(*q*)] vs ln(*q*)) (Fig. 3A) in the intermediate *q* region^50^. The calculated Porod slope (η) of -2.2 ± 0.04 (0.06 < *q* < 0.35, Fig. 3A) suggests equilibrium structure qualities of Rec1-resilin between those of Gaussian chains and collapsed polymer coils. To understand the “unfoldedness” or “random coil” likeness of Rec1-resilin in solution, the scattering data were qualitatively assessed by means of a Kratky-Debye plot^50^. The Kratky plot of Rec1-resilin (Fig. 3B & Figure 7 in Supplementary Information) displays an initial monotonic increase in the lower *q*-region, followed by a plateau with gentle negative slope in the higher *q*-region. The observed trend indicates the characteristics of a non-folded overall random coil secondary structural conformation in solution.

The Ensemble Optimization Method (EOM)^51^ is an effective tool to describe experimental SAXS data using an ensemble representation of atomic models. It allows quantitative characterization of the flexibility of a particle; and the preferential conformations of IDPs can be modelled. The use of EOM to describe experimental SAXS data for Rec1-resilin results in an extended unimodal distribution for both *R*_*g*_ and *D*_*max*_ (Fig. 4A,B). This unimodal behaviour indicates the existence of single conformational population in Rec1-resilin with an average ensemble *D*_*max*_ ~153 Å. The presented ‘pool’ (Fig. 4A) is a distribution function representing the spread of *R*_*g*_ values if the entire protein sequence were able to move through all the degrees of freedom permitted by steric and other interactions with no stable secondary structure^38^. The average pool *R*_*g*_ was determined to be ~51 Å. The ‘selected ensemble’ distribution (Fig. 4A) is the one that best fits the experimental SAXS data. The observed shift in the distribution of Rec1-resilin to smaller size (*R*_*g*_ of 47.03 Å) implies that less than 100% of the protein structure is free to move and supports the findings of some secondary structure by CD spectroscopy and partial compactness from Porod and Kratky analyses.

A typical *ab initio* reconstruction (overall external shape) of Rec1-resilin from SAXS data is illustrated in Fig. 4C. The 3D-model structure (one among an infinite ensemble of possible 3D densities) of Rec1-resilin is presented (using the GASBOR program^52^) as a chain like ensemble of dummy amino acid residues (number of residues equal to that in the protein). The dummy residues were placed anywhere in continuous space with a preferred number of close distance neighbors^51^,^52^. A Chi square (χ^2^) value of 0.58, representing the goodness of fit, was obtained for GASBOR model fits to the experimental SAXS data.

## Discussion

In this study, the use of both experimental techniques and secondary structure modelling routines reveals that Rec1-resilin in solution is an IDP with robust structural conformations that are stable over a wide range of pH and X-ray intensity. The predicted structural parameters such as the radius of gyration (*R*_*g*_), the pair-distance distribution function, P(*r*), and the Porod slope, η confirm that in aqueous solution, Rec1-resilin displays equilibrium structural features between those of Gaussian chains and collapsed polymer coils. The observations support the hypothesis that Rec1-resilin is intrinsically more compact than chemically denatured proteins yet still is qualified as an IDP. The primary structural composition of Rec1-resilin is mainly dominated by 18 copies of a 15-amino acid residue repeat sequence: GGRPSDSYGAPGGGN (Fig. 1 in Supplementary Information)^18^. In the amino acid sequence of Rec1-resilin, a Serine, *Ser* (position 5), a tyrosine, *Tyr* (position 8), a Glycine, *Gly* (position 9), and a Proline, *Pro* (position 11) are conserved in all the 18 copies. It contains a very high level of *Gly* (34.2 mol %) and *Pro* (13.8%), and it lacks hydrophobic residues with long aliphatic or aromatic side chains (Table 1 in Supplementary Information). The amino acid residue, *Gly* lacks any side chain and is highly flexible. Consequently, its presence in the peptide backbone makes ordered structures (helix, β-sheet, etc.) entropically unfavourable. On the other hand, the cyclic side chain of *Pro* (the only proteinogenic amino acid with a constrained phi angle) is too stiff to make a regular secondary structure and therefore intrinsically reduces the ability to form hydrogen bonds. The conformational constraint imposed by the presence of *Pro* residue in the peptide chain likely lowers the transition state barrier, thereby preventing secondary structure formation. Both *Gly* and *Pro* amino acid residues contribute to the propensity of the local structure to avoid folding and preclude full extension of the polypeptides^53^,^54^,^55^,^56^.

A survey of the amino acid sequences of numerous proteins possessing rubber-like elasticity (e.g. vertebrate elastins, molluscan byssus fibres, plant-derived high molecular weight glutenin, spider flagelliform silk, and spider dragline silk) revealed that the proline-glycine (*Pro*-*Gly*) motif is often-conserved. The presence of this motif has strongly been argued to be largely responsible for mediating high-amplitude bending motions and flexibility^53^. Each of these residues has distinct effects on folding and unfolding kinetics. In the case of elastin-like polypeptides (ELPs) composed of the pentapeptide repeat sequence: valine-Pro-Gly-X-Gly (X=any amino acid residue), the existence of a combined Pro-Gly threshold (2Pro+Gly > 0.6; content is expressed as a proportion of domain length) has been identified. Above this threshold value, self-interaction and amyloid formation are inhibited, leading to significant conformational disorder and enhanced backbone hydration^56^. This quantitative threshold in *Pro*-*Gly* has also been confirmed in the domains of many different ELPs including ampullate spindroin 2 (MaSp2), flagelliform silk, and elastic domains of mussel byssus thread. Amyloidogenic sequences have primarily been identified below this *Pro*-*Gly* threshold^53^,^56^. Muiznieks and Keeley^55^ experimentally investigated the specific contribution of number of *Pro* residues in ELPs and their spacing on the secondary structure and reversible self-assembly characteristics using real-time imaging. It was shown that for a combined *Pro*-*Gly* threshold of >0.6 within the hydrophobic sequences, ELPs remain substantially disordered and flexible in solution^55^. It was also shown that proline-poor regions in ELPs provide a unique contribution to assembly through localized-sheet mediated self-assembly interactions.

The combined *Pro-Gly* content in the case of Rec1-resilin has been calculated to be 0.631, which is above the threshold value hypothesized for polypeptides to display rubber-like elasticity. The analyses of the inter-*Pro* and inter-*Gly* spacings (Figures 8A & 8B in Supplementary Information) in Rec1-resilin reveal the repetitive nature of the number of residues between consecutive *Pro* and *Gly*. About 79% of inter-*Pro* spacing involves 7 or less residues (14% with 7 residue, 40% with 6 residue, and 25% with less than 6 residues) between consecutive *Pro* residues. Only 21% of inter-*Pro* spacing contains more than 8 residues indicating low content of *Pro*-free regions. The frequency of *Pro* in the peptide sequence prevents the formation of tightly packed aggregates and gives the polymer chain structural disorder, flexibility, and elasticity. It has been reported that the introduction of long *Pro*-free regions into the hydrophobic domains of ELPs promotes localized-sheet formation and decreases the ability to reversibly self associate^53^. The structural analysis of Rec1-resilin reveals that the inter-*Gly* spacing distribution (Figure 8B in Supplementary Information) is sharply peaked at low spacing, and 0, 1 or 2 residues are most preferable. The presence of non-random inter-*Pro* and inter-*Gly* spacings in the Rec1-resilin sequence appears to play a major role in the maintenance of conformational disorder and hydration that are essential to avoid amyloid formation and to achieve elastomeric properties.

The mechanism for preventing protein aggregation is traditionally discussed in terms of globular tertiary structure that promotes interfaces between polar residues and water whilst shielding non-polar residues via their burial into a hydrophobic core^53^. Both hydration and conformational disorder are fundamental requirements for rubber-like elasticity in ELPs. Examination of Rec1-resilin has indicated (1) a primary amino acid sequence that leads to disorder, (2) close positioning of the high flexibility residue (*Gly*), and (3) periodic use of the structure-breaking residue (*Pro*) in an overall hydrophilic (low hydrophobic) environment. The composition of Rec1-resilin is dominated by hydrophilic amino acid residues including both uncharged 34.2% (*Ser* 14.5%, *Thr* 1.97%, *Asn* 6.57%, *Gln* 4.24% and *Tyr* 6.9%) and charged polar 10.19% (*Asp* 3.94% and *Arg* 5.26%) hydrophilic residues (Table 1 in Supplementary Information). Consequently, the Kyte-Doolittle hydrophobicity plot of Rec1-resilin demonstrated overall hydrophilicity of the entire protein surface^19^. Water molecules form a solvation layer (bound water) around hydrophilic surface residues and have a damping effect on the attractive forces between proteins resulting in reduced protein aggregation. The close positioning of *Gly* residues along with the recurrent *Pro* residues embedded within an overall hydrophilic structural environment is thought to enable conformational plasticity and reversible switching between distinct conformational states. The presence of *Tyr* at regular spacings (Figure 8C in Supplementary Information) in Rec1-reslin offers excellent opportunity to introduce uniformly distributed *Tyr* mediated crosslinking (through di-/tri-tyrosine) to form cross-linked Rec1-resilin hydrogels^24^ that possess highly efficient elastic recoil properties necessary for reversible deformation.

Rec1-resilin, being an IDP and displaying remarkable functionalities, challenges the traditional notion that protein function depends on a unique three-dimensional structure. In-depth experimental research on IDPs and intrinsic disordered regions (IDRs) has been limited. Peng *et al.*^29^ have recently performed a comprehensive computational analysis and mapping of a large number of IDPs and IDRs (6 million proteins from 59 archaea, 471 bacterial and 110 eukaryotes and 325 viral proteomes) and observed that intrinsic disorder is abundant in each domain of biota. This investigation supports the existence of large groups of natively disordered proteins that are involved in important functions and biological roles. The IDPs were reported to lack unique 3D structures and their conformational ensembles are highly dynamic in nature^29^,^30^,^57^,^58^. The abundance of IDRs in viral proteins has also been identified, and it has been noted that viruses (e.g. HIV-1 proteins) use the IDRs not only for survival but also for aggressive invasion of the host organisms^59^. For these reasons, establishing the structure-function relationships for IDPs is coming to the forefront as a critical research challenge.

At present, there is little definitive information on the molecular basis for Rec1-resilin’s unusual multi-responsiveness in solution, and its near-perfect elasticity in the cross-linked hydrogel state. The comprehensive structural examination in this study, using a combination of in-depth computational and experimental investigations, confirms Rec1-reslin is a member of the IDP class. This work also reveals that structural features of Rec1-resilin in dilute solution inhibit protein aggregation. Kato *et al.*^60^ have recently presented compelling evidence that low-complexity proteins similar to Rec1-resilin form functional beta-sheet aggregates at high concentrations similar to those at which Rec1-resilin coacervates. Investigation of the structural assembly of Rec1-resilin at higher concentrations and in cross-linked hydrogels is the subject of a future report. It is our hope that once the conformational assemblies of Rec1-resilin in both dilute and concentrated solution and in the cross-linked hydrogel phase have been clearly identified, a comprehensive structure-function relationship for Rec1-resilin will emerge.

## Methods

### Bioinformatics

The secondary structure of Rec1-resilin was predicted from the primary amino acid sequence (Fig. 1 in Supplementary Information) using three different protein secondary structure prediction algorithms namely DSC^30^, PHDsec^31^, and SOPMA^32^. DSC predicts secondary structure from multiply-aligned homologous sequences with an overall three state prediction accuracy of 72.4%^30^. PHDsec predicts secondary structure by a system of neural networks reported with overall prediction accuracy >72% for the three states helix, strand and loop^31^. The SOPMA algorithm is based on the homologue method with overall three-state prediction accuracy of 69.5%^32^. The predictions were performed using a network protein sequence web server (http://npsa-pbil.ibcp.fr/cgi-bin/npsa_automat.pl?page=/NPSA/npsa_server.html). The naturally disordered region of Rec1-resilin was predicted from the primary amino acid sequence using the PONDR^®^ (Predictor of naturally disordered regions) algorithm^33^. PONDRs are typically feed-forward neural networks that use sequence attributes such as the fractional composition of particular amino acids, hydropathy or sequence complexity, which are averaged over these windows and the values are used to train the neural network during predictor construction. When making predictions, outputs are between 0 and 1 and are then smoothed over a sliding window of 9 amino acids^33^. The prediction was performed using a web based PONDR-FIT server (http://www.disprot.org/pondr-fit.php).

### Protein expression and purification

Synthetic Rec1-resilin construct was synthesized using a cloning strategy as reported previously^13^,^18^. Briefly, exon-1 of the *D. melanogaster* CG15920 gene was cloned and expressed as a water soluble protein in the bacteria *Escherichia coli* with a yield of ~60 mg/L of culture. The protein was then purified by a three step non-chromatographic purification method: salt precipitation (using 20% ammonium sulphate) followed by overnight dialysis at 4 °C (in excess phosphate-buffered saline, PBS) and heating at 80 °C for 10 min (with stirring). The denatured proteins were removed by centrifugation at 12,000 g for 15 min at 20 °C and the resulting protein solution in supernatant was freeze dried and stored for further analysis.

### Molecular weight determination

For molecular weight determination by sodium dodecyl sulfate-polyacrylamide gel electrophoresis (SDS-PAGE), about 50 μg of the protein was dissolved in 7.5 μL of MilliQ water and mixed with 2.5 μL of NuPAGE^®^ 4x LDS sample buffer. The prepared sample was equilibrated at 95 °C for 15 min and then loaded into a SDS-PAGE electrophoresis system made from NuPAGE^®^ Novex^®^ 4-12% Bis-Tris Protein Gel and MES SDS 1x running buffer. The protein marker was loaded into the first lane (left) and the protein into the second lane (Figure 5A in Supplementary Information). An electric potential of 140 volts was applied to the SDS-PAGE electrophoresis system for about 2 hr. The gel was then stained using Coomassie brilliant blue for 1 hr incubated on a rocking table. The gel was then gently rinsed twice with double distilled water followed by 1 hr incubation in destain solution (10% methanol and 30% acetic acid) followed by further destaining in double distilled water overnight. The gel was then gently rinsed with double distilled water and observed under UV light (Figure 5A in Supplementary Information). Compared with standard marker, the apparent molecular weight of Rec1-resilin was determined to be approximately 45 kDa by mobility on SDS-PAGE. This value is larger than the expected (theoretical) molecular weight of 28.5 kDa. This discrepancy in apparent versus predicted molecular weight of Rec1-resilin by SDS-PAGE has previously been reported^13^,^18^. Indeed, it is a property commonly observed in many cuticular proteins^61^. Therefore, matrix-assisted laser desorption-ionization time-of-flight mass spectrometry (MALDI-TOF-MS) was performed to measure the mass of the synthesized protein.

For molecular weight determination by MALDI-TOF-MS, pure protein (4.8 mg) was dissolved in 30% acetonitrile, 0.1% trifluoroacetic acid (TA30) to a final concentration of 3.0 mg/ml. 1.5 μl of matrix (sinapinic acid, saturated in ethanol), spotted onto a polished steel target plate (Bruker Daltonics, Bremen, Germany) and air dried. 2 μl of protein solution (1 pmol/μl) was mixed with 2 μl matrix (sinapinic acid, saturated in TA30). 0.5 μl of sample:matrix mix was spotted onto the previously created matrix spots and air dried. Mass spectra were acquired on an ultrafleXtreme MALDI-TOF mass spectrometer (Bruker Daltonics) operating in linear positive mode. Instrument settings were set in flexControl software (Version 3.4, Bruker Daltonics). Measurement range was set to m/z 5000 – 50,000. 5000 shots were collected for the external calibration and 20,000 shots for sample measurement. External calibration was performed using a mix of protein calibration standard I and II (Bruker Daltonics). Laser intensity and detector gain were manually adjusted for optimal resolution. The MS spectra obtained (Figure 5B in Supplementary Information) were analysed using flexAnalysis software (version 3.3, Bruker Daltonics) employing smoothing, background subtraction and peak detection algorithms.

### CD spectroscopy

For secondary structure analysis of Rec1-resilin using a CD spectrometer (J815 UV-Vis CD spectrometer, JASCO analytical instruments), a protein concentration of 100 μg/ml was used unless otherwise indicated. The desired amount of protein was dissolved in 5 mM phosphate buffer saline (PBS, Sigma Aldrich) for pH 7.4 analysis. For pH 2, 4.8 and 12 the desired amount of protein was dissolved in pH corrected (adjusted using 1 M NaOH and 1 M HCl) PBS solutions. An UV quartz cuvette with path length of 1 mm was used for all the measurements at a fixed temperature of 25 ± 0.1°C. In all the cases the detector voltage remained below 600 mV and the respective buffer background was subtracted. The online fitting program interface Dichroweb (http://dichroweb.cryst.bbk.ac.uk/html/process.shtml) was used to extract secondary structure information from CD spectra. Three different fitting algorithms namely CONTIN^35^, SELCON3^36^, and CDSSTR^37^ were used with reference spectra set 7 (containing some unordered model proteins) to extract secondary structural information.

### SAXS

The equilibrium structure and morphology of Rec1-resilin in Milli-Q Gradient A10 purified water was investigated using SAXS. Both bench-top and synchrotron SAXS beam lines were employed for in-depth understanding of the intrinsic structure. A bench-top NanoSTAR II SAXS (Bruker) with a rotating anode Cu Kα radiation source (1.541 Å) and a 2D detector was used to analyse protein concentrations of 0.1, 0.5 and 1% w/v. A scattering vector, *q* in the range of 0.005 to 0.35 Å^−1^ was used for the analysis. A synchrotron SAXS beam line with 1M Pilatus detector (utilizes an undulator source providing a very high flux to moderate scattering angles and a good flux at a total *q* range of 0.0012 to 1.1 Å^−1^) was used for low protein concentrations such as 0.013, 0.025, 0.05 and 0.1% w/v. The synchrotron SAXS resolution (extended *q* range) proves advantageous over bench-top SAXS in investigating structures of proteins in low concentrations, where there is no need for removing the effect of beam profile from the data (desmearing) as required in bench-top SAXS. In both beam lines, the samples were placed in a quartz capillary with temperature controlled at 25 ± 0.1 °C. In all the cases the buffer background was subtracted from the sample. Initially, the samples were carefully checked for any protein structural damage by X-rays (Fig. 3 in Supplementary Information); and with no damage confirmed, the scattering curves were measured at desired protein concentrations. With no effects of agglomeration or inter-particle interference observed (Fig. 4 in Supplementary Information), the scattering intensities were extrapolated to zero concentration to obtain structural information for the protein from Guinier approximation in the lower *q* region^40^, Porod analysis in higher *q* region^50^, Kratky plot of total *q* region^49^, and distance distribution function, P(*r*)^47^ for total *q* region using the PRIMUS program^41^.

With Rec1-resilin recognized to be intrinsically unfolded, the Ensemble Optimization Method (EOM) was used to fit the averaged theoretical scattering intensity from an ensemble of conformations into the experimental SAXS data^51^. A pool of *N* independent models based upon sequence and structural information was first generated. No rigid body was used in the input, and the complete random configurations of the α-carbon trace were created based upon the sequence^51^. Once the pool generation was complete, a genetic algorithm for the selection of the ensemble was performed, and the appropriate subsets of configurations fitting the experimental SAXS data were selected^38^.

## Additional Information

**How to cite this article**: Balu, R. *et al.* Structural ensembles reveal intrinsic disorder for the multi-stimuli responsive bio-mimetic protein Rec1-resilin. *Sci. Rep.* **5**, 10896; doi: 10.1038/srep10896 (2015).

## Supplementary Material

Supplementary Information

## Figures and Tables

**Table 1:** Secondary structure of Rec1-resilin predicted from modelling routines.

**Secondary structure**	**Modelling routines**		
	**DSC**^30^	**PHDsec**^31^	**SOPMA**^32^
α-helix	0%	0%	0.32%
Extended β-strand	15.48%	1.29%	1.94%
β -turn	0%	0%	8.71%
Random coil	84.52%	98.7%	89.03%

**Table 2:** CD spectrum deconvolution fit parameters.

**pH**	**Algorithm**	**Helix**	**Sheets and turn**	**Unordered**
2	CONTIN^35^	0.060	0.395	0.545
4.8		0.054	0.414	0.532
7.4		0.058	0.396	0.546
12		0.055	0.411	0.535
2	SELCON3^36^	0.024	0.34	0.629
4.8		0.020	0.318	0.606
7.4		0.029	0.384	0.573
12		0.029	0.389	0.573
2	CDSSTR^37^	0.010	0.410	0.560
4.8		0.010	0.420	0.550
7.4		0.010	0.410	0.550
12		0.010	0.401	0.550

## References

[b1] Gomes S., Leonor I. B., Mano J. F., Reis R. L. & Kaplan D. L. Natural and genetically engineered proteins for tissue engineering. Prog. Polym. Sci. 37, 1–17 (2012). 22058578 10.1016/j.progpolymsci.2011.07.003 PMC3207498

[b2] DiMarco R. L. & Heilshorn S. C. Multifunctional materials through modular protein engineering. Adv. Mater. 24, 3923–3940 (2012). 22730248 10.1002/adma.201200051

[b3] Balu R., Whittaker J., Dutta N. K., Elvin C. M. & Choudhury N. R. Multi-responsive biomaterials and nanobioconjugates from resilin-like protein polymers. J. Mater. Chem. B. 2, 5936–5947 (2014). 10.1039/c4tb00726c 32261846

[b4] Amiram M., Quiroz F. G., Callahan D. J. & Chilkoti A. A highly parallel method for synthesizing DNA repeats enables the discovery of ‘smart’ protein polymers. Nature Mater. 10, 141–148 (2011). 21258353 10.1038/nmat2942 PMC3075872

[b5] Tamerler C. & Sarikaya M. Genetically designed peptide-based molecular materials. ACS Nano 3, 1606–1615 (2009). 21452861 10.1021/nn900720g

[b6] Kushner A. M. & Guan Z. Modular design in natural and biomimetic soft materials. Angew. Chem. Int. Ed. 50, 9026–9057 (2011). 10.1002/anie.201006496 21898722

[b7] Tsien R. Y. Constructing and exploiting the fluorescent protein paintbox (Nobel lecture). Angew. Chem. Int. Ed. 48, 5612–5626 (2009). 10.1002/anie.200901916 19565590

[b8] Nettles D. L., Chilkoti A. & Setton L. A. Applications of elastin-like polypeptides in tissue engineering. Adv. Drug Deliv. Rev. 62, 1479–1485 (2010). 20385185 10.1016/j.addr.2010.04.002 PMC2935943

[b9] Balu R. *et al.* An16-resilin: An advanced multi-stimuli responsive resilin-mimetic protein polymer. Acta Biomater. 10, 4768–4777 (2014). 25107894 10.1016/j.actbio.2014.07.030

[b10] Su R. S. C., Renner J. N. & Liu J. C. Synthesis and characterization of recombinant abductin-based proteins. Biomacromolecules 14, 4301–4308 (2013). 24147646 10.1021/bm401162g

[b11] Lyons R. E. *et al.* Molecular and functional characterisation of resilin across three insect orders. Insect Biochem. Mol. Biol. 41, 881–890 (2011). 21878390 10.1016/j.ibmb.2011.08.002

[b12] Ardell D. H. & Andersen S. O. Tentative identification of a resilin gene in *Drosophila melanogaster*. Insect Biochem. Mol. Biol. 31, 965–970 (2001). 11483432 10.1016/s0965-1748(01)00044-3

[b13] Lyons R. E. *et al.* Design and facile production of recombinant resilin-like polypeptides: gene construction and a rapid protein purification method. Protein Eng. Des. Sel. 20, 25–32 (2007). 17218334 10.1093/protein/gzl050

[b14] Renner J. N., Cherry K. M., Su R. S. C. & Liu J. C. Characterization of resilin-based materials for tissue engineering applications. Biomacromolecules 13, 3678–3685 (2012). 23057410 10.1021/bm301129b

[b15] McGann C. L., Levenson E. A. & Kiick K. L. Resilin-based hybrid hydrogels for cardiovascular tissue engineering. Macromol. Chem. Phys. 214, 203–213 (2013). 10.1002/macp.201200412 PMC3744378 23956463

[b16] Li L., Tong Z., Ji X. & Kiick K. L. Resilin-like polypeptide hydrogels engineered for versatile biological functions. Soft Matter 9, 665–673 (2013). 23505396 10.1039/C2SM26812D PMC3595062

[b17] Whittaker J., Balu R., Choudhury N. R. & Dutta N. K. Biomimetic protein‐based elastomeric hydrogels for biomedical applications. Polym. Int. 63, 1545–1557 (2014).

[b18] Elvin C. M. *et al.* Synthesis and properties of crosslinked recombinant pro-resilin. Nature 437, 999–1002 (2005). 16222249 10.1038/nature04085

[b19] Dutta N. K. *et al.* A genetically engineered protein responsive to multiple stimuli. Angew. Chem. Int. Ed. 50, 4428–4431 (2011). 10.1002/anie.201007920 21472931

[b20] Dutta N. K. *et al.* Physical approaches for fabrication of organized nanostructure of resilin-mimetic elastic protein Rec1-resilin. Biomaterials 30, 4868–4876 (2009). 19592086 10.1016/j.biomaterials.2009.06.019

[b21] Truong M. Y. *et al.* A pH-responsive interface derived from resilin-mimetic protein Rec1-resilin. Biomaterials 31, 4434–4446 (2010). 20223516 10.1016/j.biomaterials.2010.02.019

[b22] Mayavan S. *et al.* Self-organization, interfacial interaction and photophysical properties of gold nanoparticle complexes derived from resilin-mimetic fluorescent protein Rec1-resilin. Biomaterials 32, 2786–2796 (2011). 21295342 10.1016/j.biomaterials.2010.12.030

[b23] Dutta N. K. *et al.*, inventors; University of South Australia, assignee. Template directed formation of metal nanoparticles and uses thereof. International patent WO 2,014,071,463A1. 2014 May 15.

[b24] Truong M. Y. *et al.* The effect of hydration on molecular chain mobility and the viscoelastic behavior of resilin-mimetic protein-based hydrogels. Biomaterials 32, 8462–8473 (2011). 21868089 10.1016/j.biomaterials.2011.07.064

[b25] Tamburro A. M. *et al.* Molecular and supramolecular structural studies on significant repetitive sequences of resilin. ChemBioChem 11, 83–93 (2010). 19943267 10.1002/cbic.200900460

[b26] Nairn K. M. *et al.* A synthetic resilin is largely unstructured. Biophys. J. 95, 3358–3365 (2008). 18586853 10.1529/biophysj.107.119107 PMC2547447

[b27] Elliott G. F., Huxley A. F. & Weis-Fogh T. On the structure of resilin. J. Mol. Biol. 13, 791–795 (1965).

[b28] Uversky V. N. A decade and a half of protein intrinsic disorder: biology still waits for physics. Protein Sci. 22, 693–724 (2013). 23553817 10.1002/pro.2261 PMC3690711

[b29] Peng Z. *et al.* Exceptionally abundant exceptions: comprehensive characterization of intrinsic disorder in all domains of life. Cell Mol. Life Sci. 72, 137–151 (2015). 24939692 10.1007/s00018-014-1661-9 PMC11113594

[b30] King R. D. & Sternberg M. J. E. Identification and application of the concepts important for accurate and reliable protein secondary structure prediction. Protein Sci. 5, 2298–2310 (1996). 8931148 10.1002/pro.5560051116 PMC2143286

[b31] Rost B. & Sander C. Prediction of protein secondary structure at better than 70% accuracy. J. Mol. Biol. 232, 584–599 (1993). 8345525 10.1006/jmbi.1993.1413

[b32] Geourjon C. & Deleage G. SOPMA: significant improvements in protein secondary structure prediction by consensus prediction from multiple alignments. Comput. Appl. Biosci. 11, 681–684 (1995). 8808585 10.1093/bioinformatics/11.6.681

[b33] Xue B., DunBrack R. L., Williams R. W., Dunker A.K. & Uversky V. N. PONDR-Fit: A meta-predictor of intrinsically disordered amino acids. Biochim. Biophys. Acta , 1804, 996–1010 (2010). 20100603 10.1016/j.bbapap.2010.01.011 PMC2882806

[b34] Gokce I., Woody R. W., Anderluh G. & Lakey J. H. Single peptide bonds exhibit poly(Pro)II (“Random coil”) circular dichroism spectra. J. Am. Chem. Soc. 127, 9700–9701 (2005). 15998070 10.1021/ja052632x

[b35] Stokkum I. H. M. V., Spoelder H. J. W., Bloemendal M., Grondelle R. & Groen F. C. A. Estimation of protein secondary structure and error analysis from circular dichroism spectra. Anal Biochem. 191, 110–118 (1990). 2077933 10.1016/0003-2697(90)90396-q

[b36] Sreerama N. & Woody R. W. A self-consistent method for the analysis of protein secondary structure from circular dichroism. Anal Biochem. 209, 32–44 (1993). 8465960 10.1006/abio.1993.1079

[b37] Johnson W. C. Analyzing protein circular dichroism spectra for accurate secondary structures. Proteins: Struct. Funct. Bioinf. 35, 307–312 (1999). 10328265

[b38] Bernado P. & Svergun D. I. Structual analysis of intrinsically disordered proteins by small‐angle X ‐ray scattering. Mol. Biosyst. 8, 151–167 (2012). 21947276 10.1039/c1mb05275f

[b39] Brechot V. R. & Durand D. How random are intrinsically disordered proteins? A small angle scattering perspective. Curr. Protein Pept. Sci. 13, 55–75 (2012). 22044150 10.2174/138920312799277901 PMC3394175

[b40] Lipfert J. & Doniach S. Small-angle X-ray scattering from RNA, proteins, and protein complexes. Annu. Rev. Biophys. Biomol. Struct. 36, 307–327 (2007). 17284163 10.1146/annurev.biophys.36.040306.132655

[b41] Konarev P. V., Volkov V. V., Sokolova A. V., Koch M. H. J. & Svergun D. I. PRIMUS: a Windows PC-based system for small-angle scattering data analysis. J. Appl. Cryst. 36, 1277–1282 (2003).

[b42] Mylonas E. & Svergun D. I. Accuracy of molecular mass determination of proteins in solution by small-angle X-ray scattering. J. Appl. Cryst. 40, 245–249 (2007).

[b43] Receveur-Bréchot V. & Durand D. How random are intrinsically disordered proteins? A small angle scattering perspective, Curr. Protein Pept. Sci. 13, 55–75 (2012). 22044150 10.2174/138920312799277901 PMC3394175

[b44] Permyakov S., Millett I., Doniach S., Permyakov E. & Uversky V. Natively unfolded C-terminal domain of caldesmon remains substantially unstructured after the effective binding to calmodulin. Proteins 53, 855–62 (2003). 14635127 10.1002/prot.10481

[b45] Kohn J. E. *et al.* Random-coil behavior and the dimensions of chemically unfolded proteins. Proc. Natl. Acad. Sci. U.S.A. 101, 12491–12496 (2004). 15314214 10.1073/pnas.0403643101 PMC515087

[b46] Narang P., Bhushan K., Bose S. & Jayaram B. A computational pathway for bracketing native-like structures for small alpha helical globular proteins. Phys. Chem. Chem. Phys. 7, 2364–2375 (2005). 19785123 10.1039/b502226f

[b47] Feigin L. A. & Svergun D. I. Structure analysis by small-angle X-ray and neutron scattering (ed. Taylor G. W. , Plenum Press, New York, 1987).

[b48] Bernado P. Effect of interdomain dynamics on the structure determination of modular proteins by small-angle scattering. Eur. Biophys. J. 39, 769–80 (2010). 19844700 10.1007/s00249-009-0549-3

[b49] Putnam C. D., Hammel M., Hura G. L. & Tainer J. A. X-ray solution scattering (SAXS) combined with crystallography and computation: defining accurate macromolecular structures, conformations and assemblies in solution. Q. Rev. Biophys. 40, 191–285 (2007). 18078545 10.1017/S0033583507004635

[b50] Porod G. Small-angle X-ray scattering (eds. Glatter O. & Kratky O. , Academic Press, London, 1982).

[b51] Bernado P., Mylonas E., Petoukhov M. V., Blackledge M. & Svergun D. I. Structural characterization of flexible proteins using small-angle X-ray scattering. J. Am. Chem. Soc. 129, 5656–5664 (2007). 17411046 10.1021/ja069124n

[b52] Svergun D. I., Petoukhov M. V. & Koch M. H. J. Determination of domain structure of proteins from X-ray solution scattering. Biophys. J. 80, 2946–2953 (2001). 11371467 10.1016/S0006-3495(01)76260-1 PMC1301478

[b53] Rausche S. & Pomès R. Fuzziness: Structural disorder in protein complexes (eds. Tompa P. & Fuxreiter M. , Springer, Texas, 2011).

[b54] Eyles S. J. & Gierasch L. M. Multiple roles of prolyl residues in structure and folding. J. Mol. Biol. 301, 737–747 (2000). 10966780 10.1006/jmbi.2000.4002

[b55] Muiznieks L. D. & Keeley F. W. Proline periodicity modulates the self-assembly properties of elastin-like polypeptides. J. Biol. Chem. 285, 39779–39789 (2010). 20947499 10.1074/jbc.M110.164467 PMC3000959

[b56] Rauscher S., Baud S., Miao M., Keeley F. W. & Pomès R. Proline and glycine control protein self-organization into elastomeric or amyloid fibrils. Structure 14, 1667–1676 (2006). 17098192 10.1016/j.str.2006.09.008

[b57] Babu M. M., Lee R. V., Groot N. S. & Gsponer J. Intrinsically disordered proteins: regulation and disease. Curr. Opin. Struct. Biol. 21, 1–9 (2011). 21514144 10.1016/j.sbi.2011.03.011

[b58] Ruskamo S. *et al.* Juxtanodin is an intrinsically disordered F-actin-binding protein. Sci. Rep. 2, 899; 10.1038/srep00899 (2012). 23198089 PMC3509349

[b59] Marcin B. X., Mizianty J., Kurgan L. & Uversky V. N. Protein intrinsic disorder as a flexible armor and a weapon of HIV-1 Cell. Mol. Life Sci. 69, 1211–1259 (2012). 10.1007/s00018-011-0859-3 PMC11114566 22033837

[b60] Kato M. *et al.* Cell-free formation of RNA granules: low complexity sequence domains form dynamic fibers within hydrogels. Cell 11, (149), 753–767 (2012). 10.1016/j.cell.2012.04.017 PMC6347373 22579281

[b61] Andersen S. O., Hørjup P. & Roepstorff P. Insect cuticular proteins. Insect Biochem. Mol. Biol. 25, 153–176 (1995). 7711748 10.1016/0965-1748(94)00052-j

