# Supplementary material for: Structural ensembles reveal intrinsic disorder for the multi-stimuli responsive bio-mimetic protein Rec1-resilin
Source: Sci Rep. 2015 Jun 4;5:10896. doi: 10.1038/srep10896 (PMC4455251; doi:10.1038/srep10896)
Supplement: Supplementary Information [file srep10896-s1.doc]

**Supplementary information**

Structural ensembles reveal intrinsic disorder for the multi-stimuli responsive bio-mimetic protein Rec1-resilin

Rajkamal Balu^1^, Robert Knott^2^, Nathan P. Cowieson^3^, Christopher M. Elvin^4^, Anita J. Hill^5^, Namita R. Choudhury^1*^ & Naba K. Dutta^1*^

^1^Ian Wark Research Institute, University of South Australia, Mawson Lakes campus, Mawson Lakes, South Australia 5095, Australia

^2^ANSTO, Private Mail Bag, Kirrawee, New South Wales 2232, Australia

^3^Centre for Synchrotron Science, Monash University, Victoria 3800, Australia

^4^CSIRO Agriculture, Level 6, Queensland Bioscience Precinct, St Lucia, Queensland 4067, Australia

^5^CSIRO Manufacturing, Clayton, Victoria 3168, Australia

**E-mail address*: naba.dutta@unisa.edu.au; namita.choudhury@unisa.edu.au

**Tel*.: +61-883023546

**Fax*: +61-883023683

**URL*: <http://www.unisa.edu.au/research/ian-wark-research-institute/>

**Table 1.** Amino acid composition of Rec1-resilin. The composition does not include the histidine tag segment.

| **Rec1-resilin** | **Amino acid** | **Code** | **No. of Units** | **Mol %** |
| --- | --- | --- | --- | --- |
| **Non-polar side chain** | Glycine | Gly/G | 104 | 34.2 |
|  | Alanine | Ala/A | 19 | 6.25 |
|  | Valine | Val/V | 1 | 0.33 |
|  | Leucine | Leu/L | 2 | 0.66 |
|  | Isoleucine | Ile/I | - | - |
|  | Methonine | Met/M | - | - |
|  | Proline | Pro/P | 42 | 13.81 |
|  | Phenylalaline | Phe/F | 1 | 0.33 |
|  | Tryptophan | Try/W | - | - |
| **Total nonpolar** | |  | **169** | **55.59** |
| **Uncharged polar side chain** | Serine | Ser/S | 44 | 14.47 |
|  | Threonine | Thr/T | 6 | 1.97 |
|  | Aspargine | Asn/N | 20 | 6.57 |
|  | Glutamine | Gln/Q | 13 | 4.24 |
|  | Tyrosine | Tyr/Y | 21 | 6.9 |
|  | Cysteine | Cys/C | - | - |
| **Total uncharged polar** | |  | **104** | **34.21** |
| **Charge polar side chain** | Lysine | Lys/K | 2 | 0.66 |
|  | Arginine | Arg/R | 16 | 5.26 |
|  | Histidine | His/H | - | - |
|  | Aspartic acid | Asp/D | 12 | 3.94 |
|  | Glutamic acid | Glu/E | 1 | 0.33 |
| **Total Charged Polar Side Chain** | |  | **31** | **10.19** |
| **Total** | | | **304** | **100** |


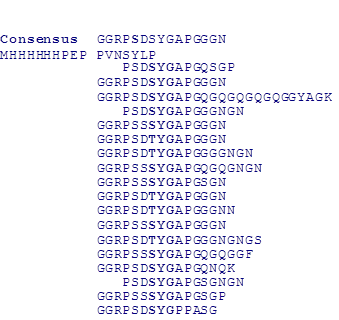


**Figure 1.** Structural consensus and alignment of primary amino acid repeat sequence in Rec1-resilin. Single-letter code is used to represent amino acids.


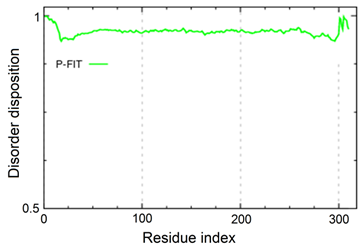


**Figure 2.** Plot of disordered regions of Rec1-resilin as a function of primary amino acid residue index, predicted using PONDR-FIT algorithm^1^. All regions of Rec1-resilin display unordered (disordered) structure with disordered disposition above a threshold of 0.5.


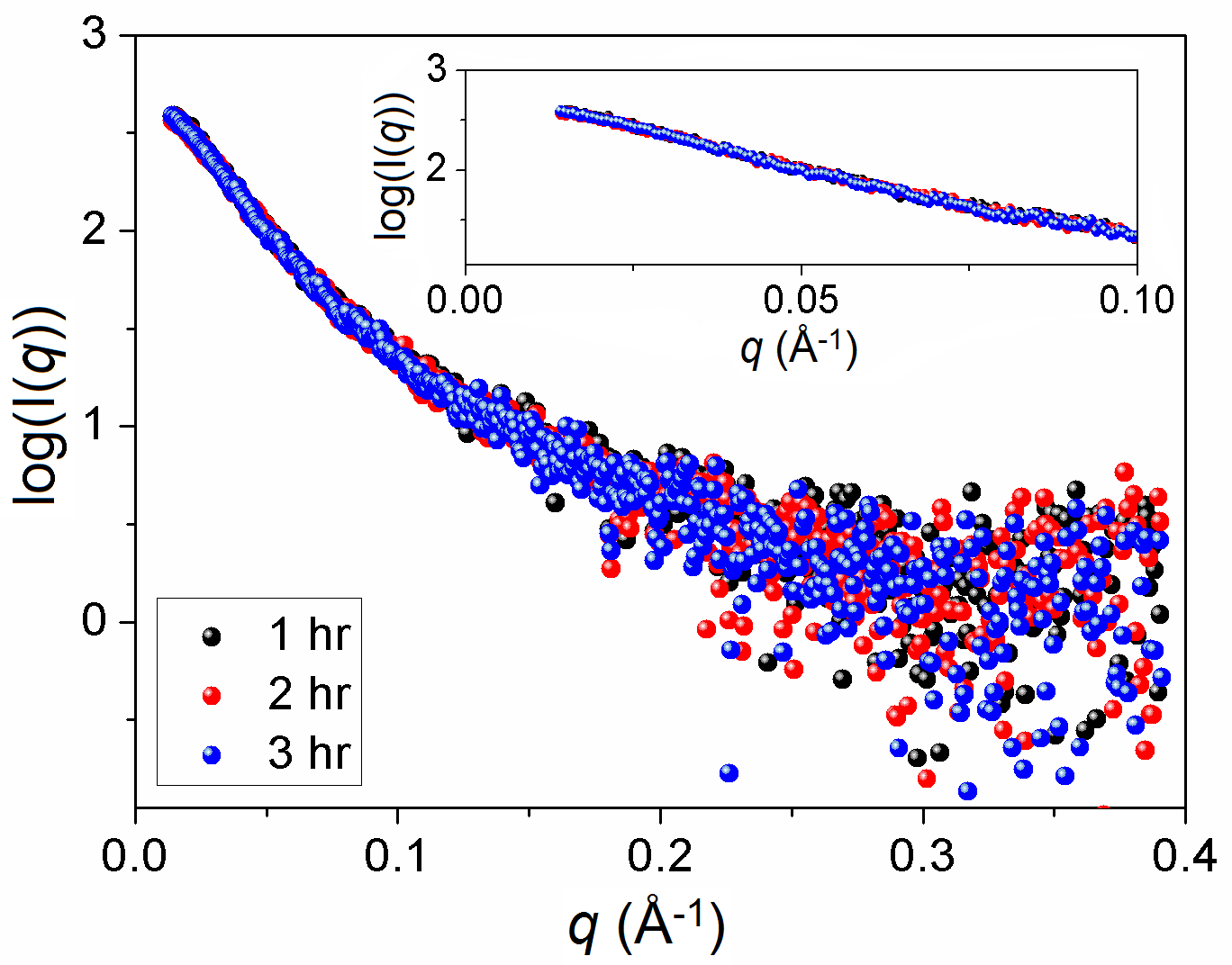


**Figure 3.** Effect of SAXS radiation time on scattering pattern of 1% Rec1-resilin*.* Inset is the zoom of lower *q* region showing consistency in data. No change in the scattering cross section with time confirms stability of the protein conformation and no radiation damage over time.


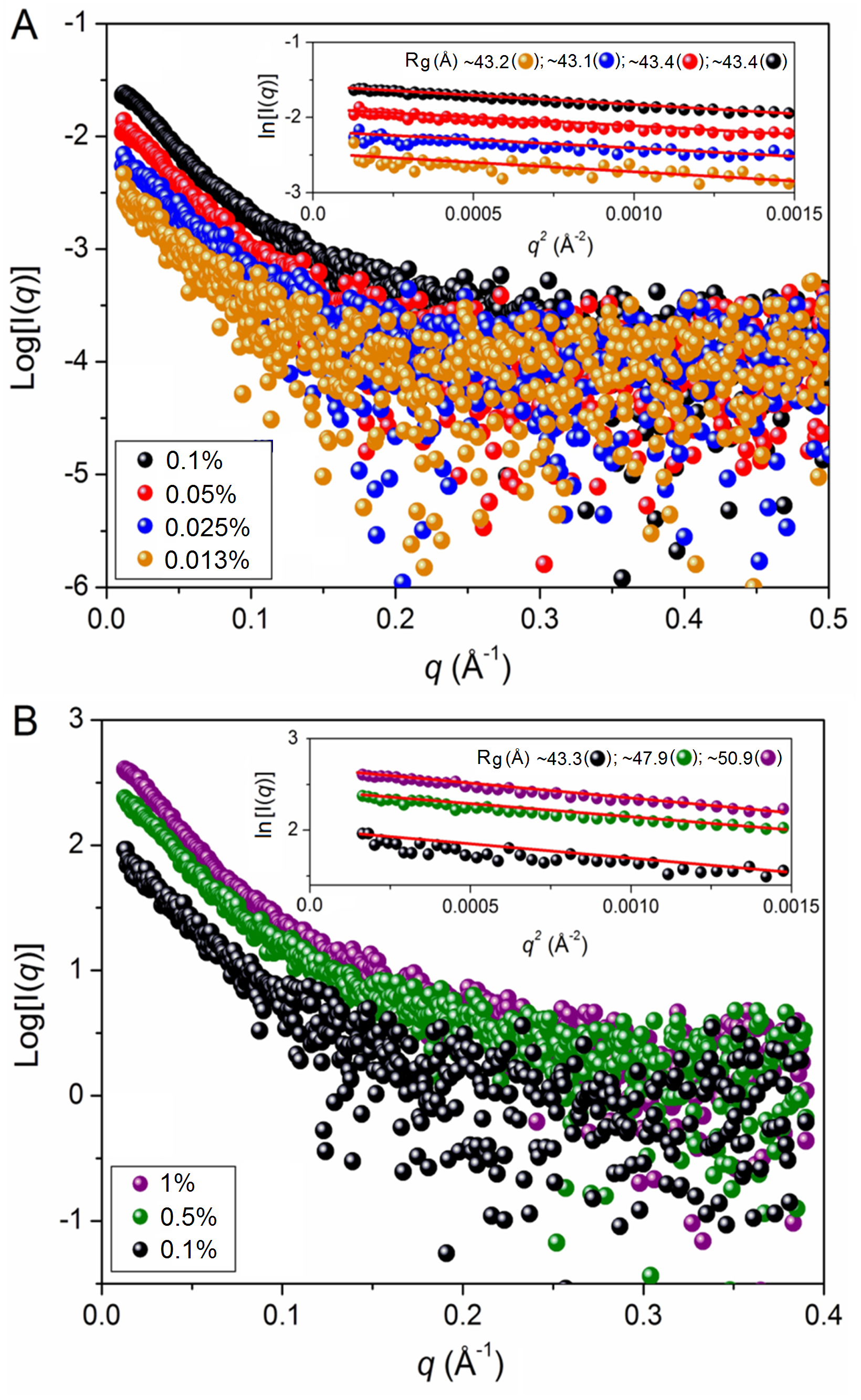


**Figure 4.** SAXS cross section of Rec1-resilin in aqueous solution as a function of concentration. (A) Synchrotron SAXS: Yellow, 0.013%; Blue, 0.025%; Red, 0.05%; Black 0.1%. The insets are the corresponding Guinier approximation plot with calculated radius of gyration (*R_g_*), using PRIMUS program^2^ (B) Bench-top SAXS: Black, 0.1%; Green, 0.5%; Purple, 1%. The insets are the corresponding Guinier approximation plot with calculated radius of gyration (*R_g_*), using PRIMUS program^2^.


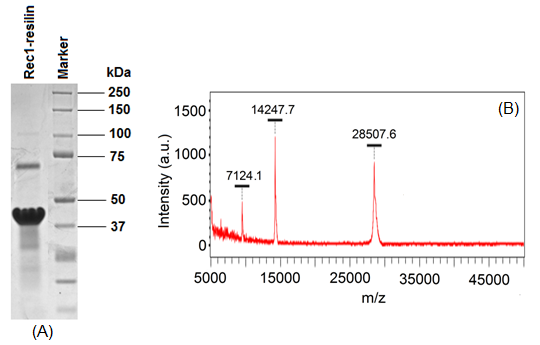


**Figure 5.** (A) Sodium dodecyl sulfate polyacrylamide gel electrophoresis (SDS-PAGE) and (B) Matrix assisted laser desorption ionization time-of-flight (MALDI-TOF) mass spectroscopy results of synthesized Rec1-resilin. The three m/z species (right to left) detected in mass spectra are the [M+H]^+^, [M+2H]^2+^, and [M+4H]^4+^ species of Rec1-resilin.

*
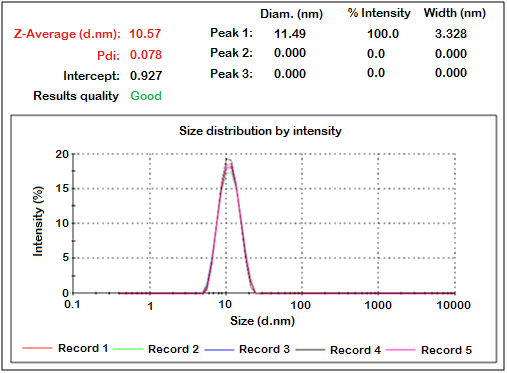
*

**Figure 6.** Hydrodynamic size measurement of 0.1% Rec1-resilin in water using dynamic light scattering (DLS) technique*.* The peak shows the size distribution of protein by intensity of light scattering over five measurements. The hydrodynamic diameter (*D_h_*) of the protein particle is observed to be 10.57 nm.

*
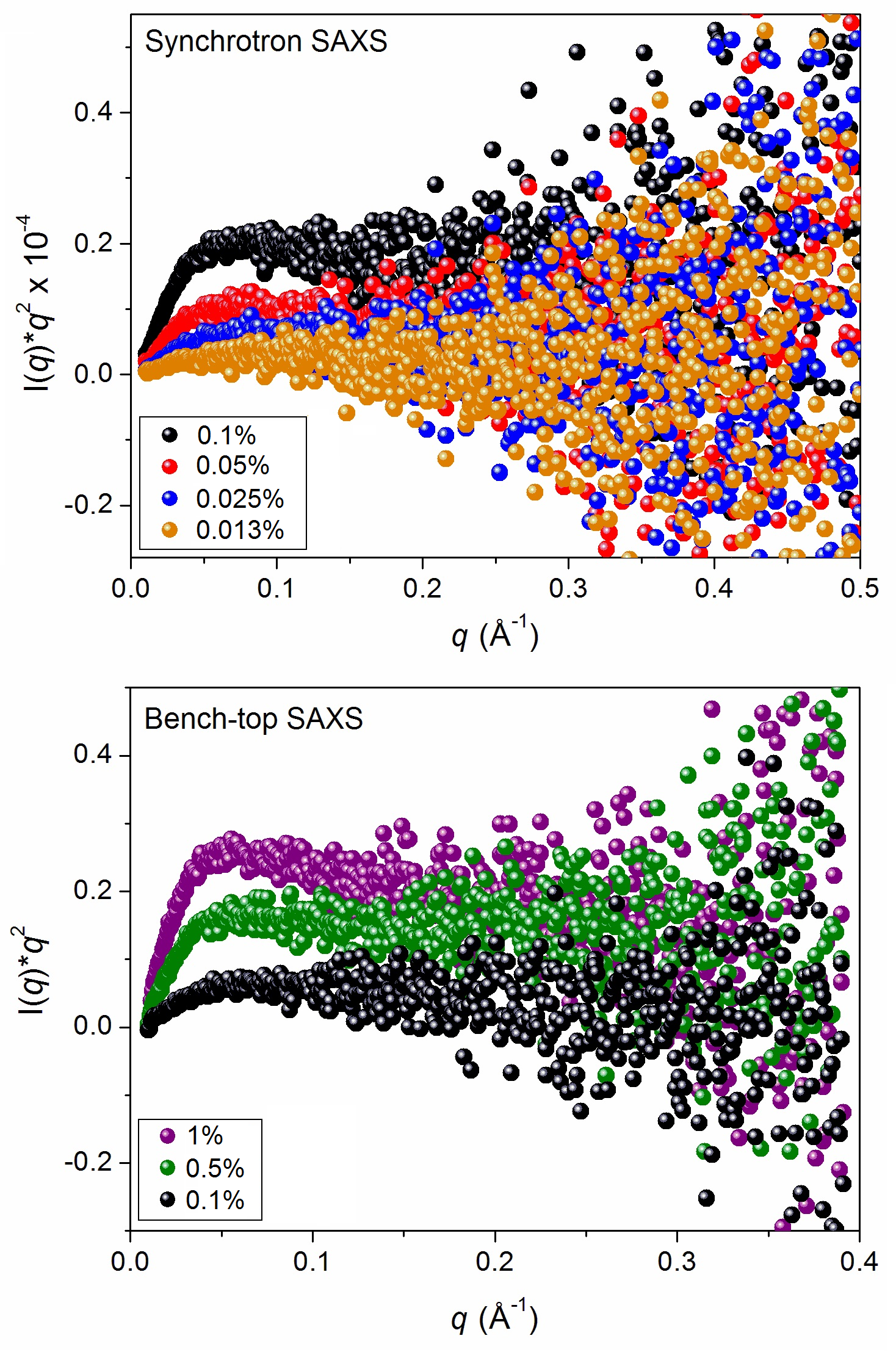
*

**Figure 7.** Kratky plot of Rec1-resilin in aqueous solution derived using synchrotron SAXS (top) and bench-top SAXS (bottom) data. The Kratky plot displays an initial monotonic increase in the lower *q*-region, followed by a plateau with gentle negative slope in the higher *q*-region. The observed trend indicates the characteristics of a non-folded overall random coil secondary structural conformation in solution.


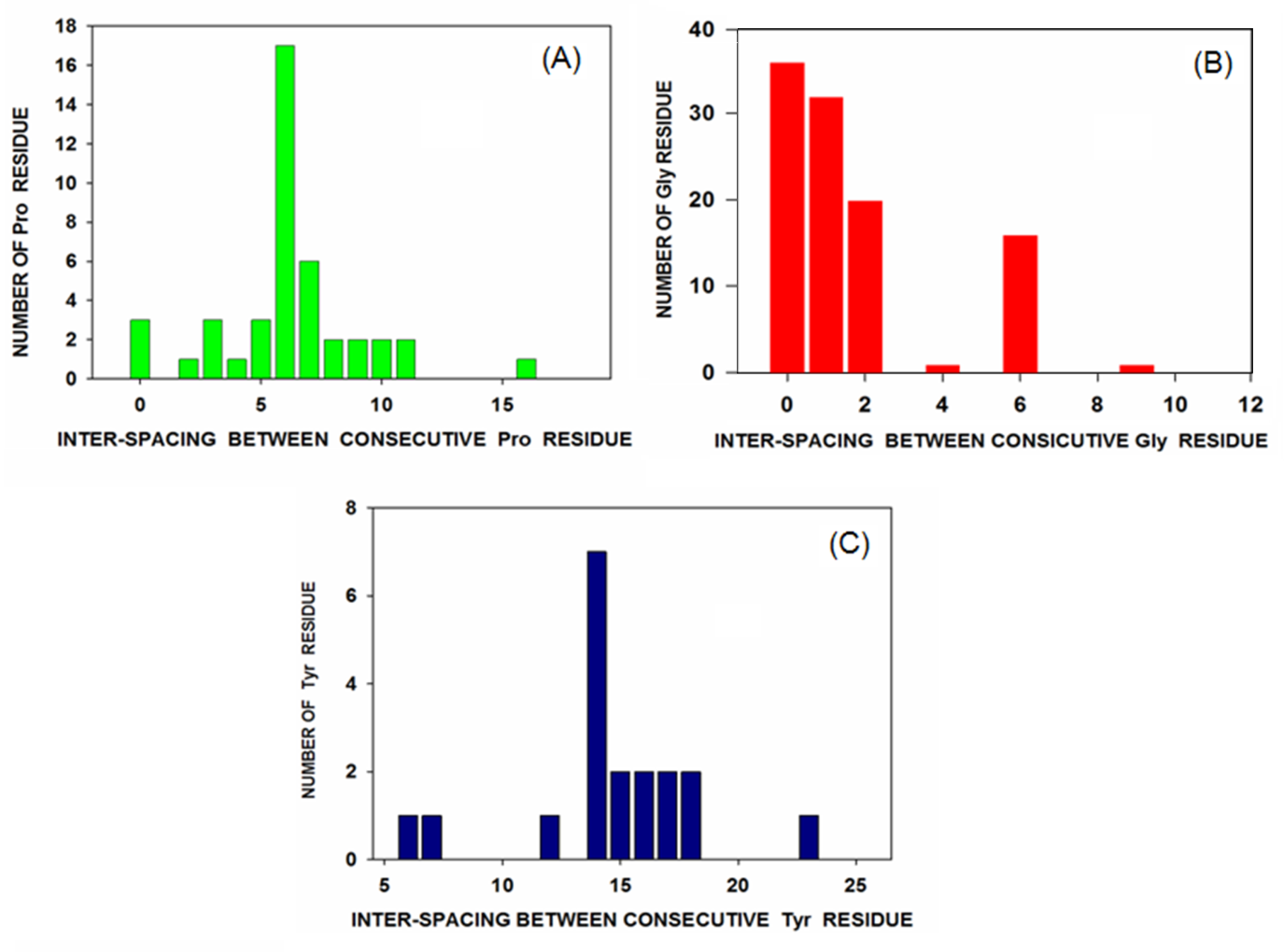


**Figure 8.** Plot of the periodicity of the amino acid residues (A) Proline (*Pro*), (B) Glycine (*Gly*), and (C) Tyrosine (*Tyr*) in Rec1-resilin.

**References**

1. Xue, B., Dunbrack, R. L., Williams, R. W., Dunker, A. K. & Uversky, V. N. PONDR-Fit: A meta-predictor of intrinsically disordered amino acids. *Biochim. Biophys. Acta.* **1804**, 996-1010 (2010).
2. Konarev, P. V., Volkov, V. V., Sokolova, A. V., Koch, M. H. J. & Svergun, D. I. PRIMUS: a Windows PC-based system for small-angle scattering data analysis. *J. Appl. Cryst*. **36**, 1277-1282 (2003).
